# Supplementary material for: Zinc-Induced Folding and Solution Structure of the Eponymous Novel Zinc Finger from the ZC4H2 Protein
Source: Biomolecules. 2025 Jul 28;15(8):1091. doi: 10.3390/biom15081091 (PMC12383448; doi:10.3390/biom15081091)
Supplement: Supplementary file 1 [file biomolecules-15-01091-s001.zip › biomolecules-3742906-supplementary.pdf]

Supporting Material for

Zinc-induced folding and solution structure of the eponymous novel zinc finger from the ZC4H2 protein

Rilee E. Harris, Antonio J. Rua, and Andrei T. Alexandrescu*

Department of Molecular and Cell Biology, University of Connecticut, Storrs, CT 06269, USA

Table of contents:

Figure S1 – DOSY spectrum of ZC4H2-ZL
Figure S2 – ^{13}C- and ^{15}N-HSQC spectra of ZL-Zn^{2+} with assigned fingerprint regions
Figure S3 –^{1}H NMR spectra of ZS and ZL in the presence and absence of zinc
Figure S4 – Thermal unfolding of ZL-Zn^{2+} by NMR
Figure S5 – Histidine pH titrations for the unfolded ZL fragment without zinc
Figure S6 – NMR secondary structure and NOEs supporting a short C-terminal α-helix
Figure S7 – NMR fails to detect interaction between ZC4H2-ZL and ubiquitin

Table S1 – Statistics for the 20 best NMR structures of the ZL fragment.

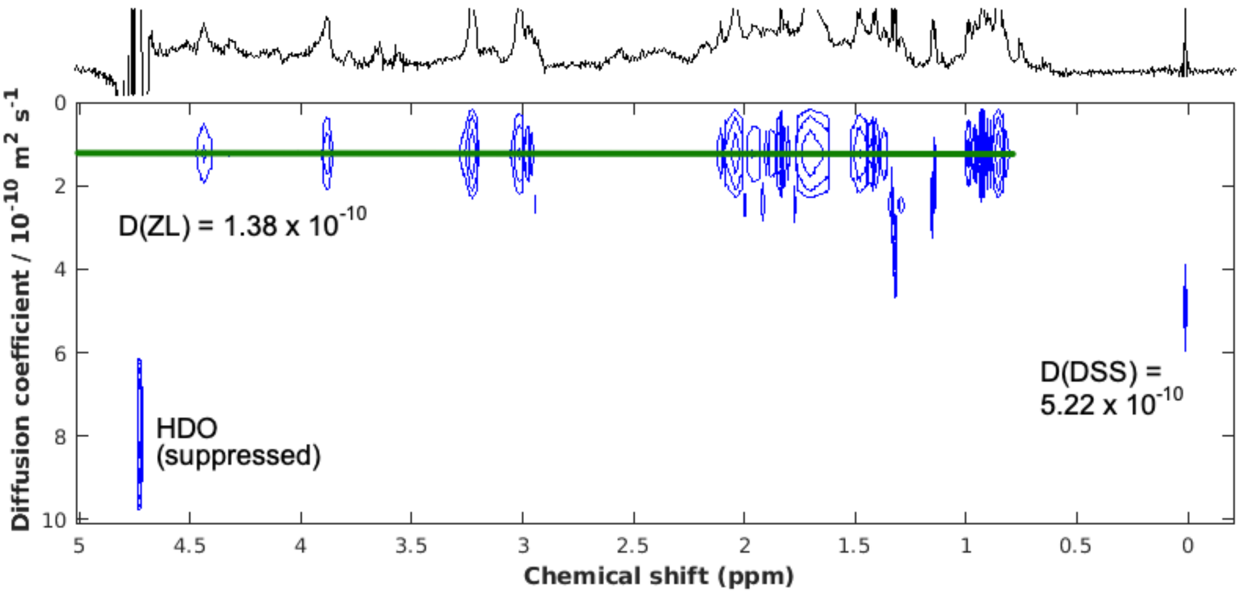

Supplementary Figure S1 DOSY spectrum of ZC4H2-ZL. A 0.3 mM ZL sample in D_2O at pH 6.87 with equimolar $ZnCl_2$ was used for the experiments. Although most NMR work for ZL was done at a 10 C, a temperature of 25 C was used for the DOSY experiments to avoid convection artefacts. NMR diffusion data were acquired on a 600 MHz Varian Inova spectrometer with the *Doneshot* pulse sequence and processed with the GNAT program on the NMRbox platform. As indicated in the figure, diffusion coefficients $D_{DSS} = 5.22 \times 10^{-10}$ m^2 s^{-1} and $D_{ZL} = 1.38 \times 10^{-10}$ m^2 s^{-1} were obtained for the DSS standard and ZL-Zn^{2+}, respectively. From the R_h (radius of hydration) value of 3.34 Å for DSS and the formula $R_{h,\,ZL} = (D_{DSS}/D_{Z0})*R_{h,DSS}$, an Rh value of 12.6 Å was calculated for the folded Zn^{2+}-bound ZL peptide, consistent with a monomer (an R_h of 12.35 Å is expected for a 27-residue monomer, compared to 15.10 Å for a 54-residue dimer).

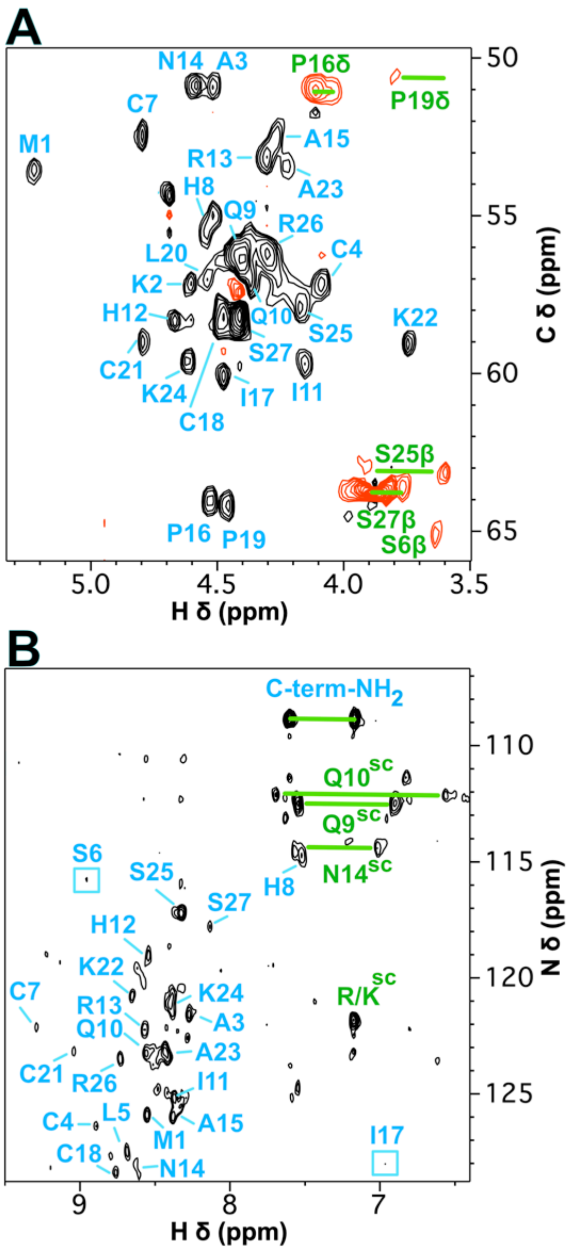

Supplementary Figure S2 ^{13}C- and ^{15}N-HSQC spectra of ZL-Zn^{2+} with assigned fingerprint regions. The ^{1}H-^{13}C (**A**) and ^{1}H-^{15}N HSQC (**B**) spectra were collected on samples at natural isotope abundance. Backbone Hα-Cα and H-N crosspeaks are labeled with their sequence-specific NMR assignments in blue, correlations due to sidechains in green. The multiplicity-edited ^{1}H-^{13}C HSQC in (A) was recorded on a Bruker 600 MHz Neo spectrometer equipped with a cryoprobe on a 0.9 mM ZL sample with equimolar $ZnCl_2$ dissolved in D_2O. In the multiplicity-edited ^{1}H-^{13}C HSQC spectrum, CH and CH_3 groups give black contours, whereas CH_2 groups red contours The ^{1}H-^{15}N HSQC in (B) was recorded on a Bruker 800 MHz Neo spectrometer with a cryoprobe on a 1.6 mM ZL sample dissolved in H_2O, at pH 5.9 and a temperature of 10 C in a total acquisition time of 12 h. Because of the low sensitivity of the ^{1}H-^{15}N HSQC at natural abundance, assignments of weak peaks in the spectrum should be considered tentative.

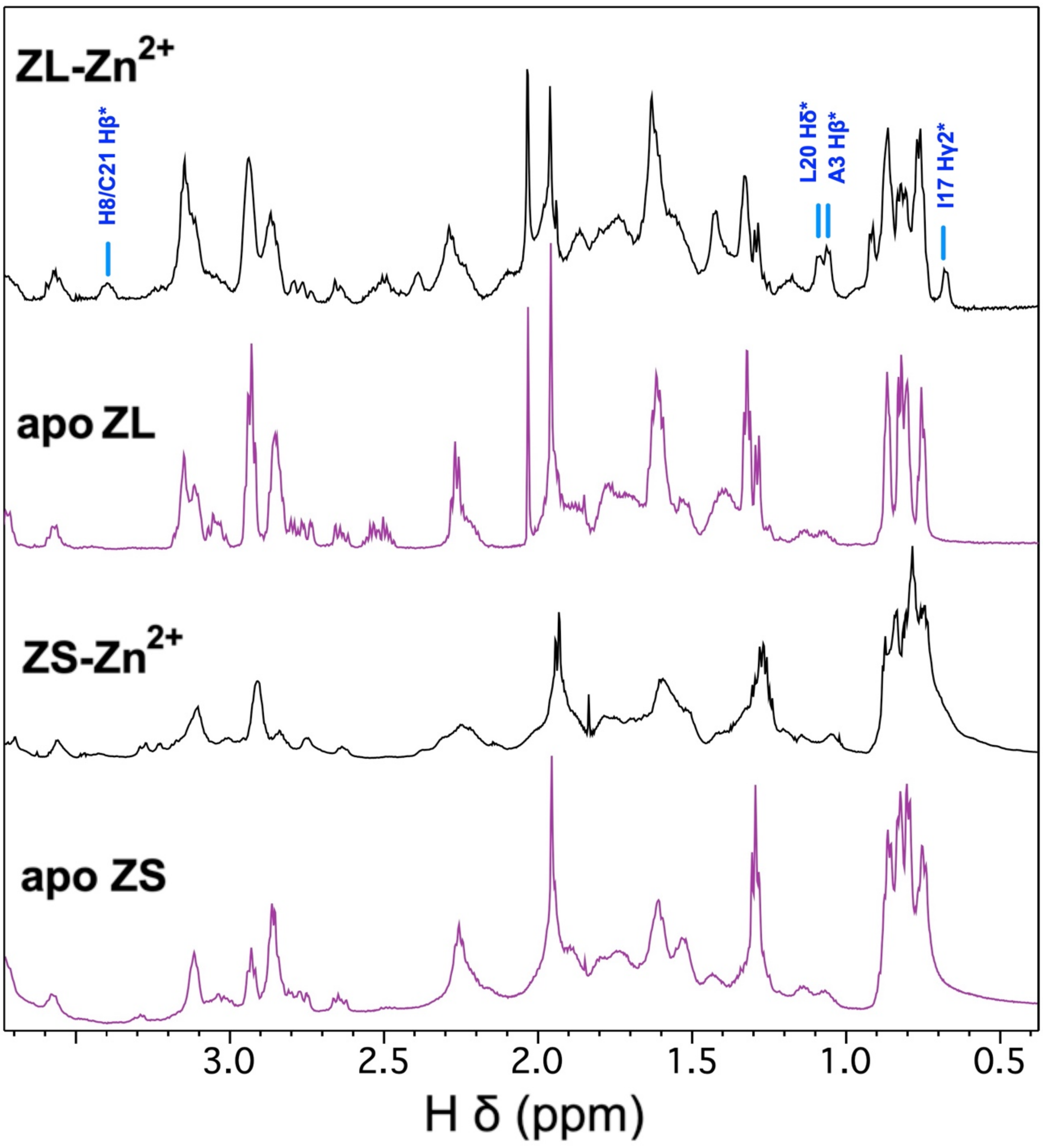

Supplementary Figure S3 ^{1}H NMR spectra of ZS and ZL in the presence and absence of zinc. The aliphatic regions of spectra correspond to those in Fig. 2A and are shown to illustrate that the NMR line-broadening and the poor chemical shift dispersion of ZS in the presence of zinc is due to dynamic heterogeneity rather than fast hydrogen exchange of labile amide protons, since NMR signals from non-exchangeable carbon-bound protons are also affected. Some of the resolved resonances in the zinc-bound ZL fragment are labeled. Note that these resonances are broadened in the spectrum of the zinc-bound ZS fragment.

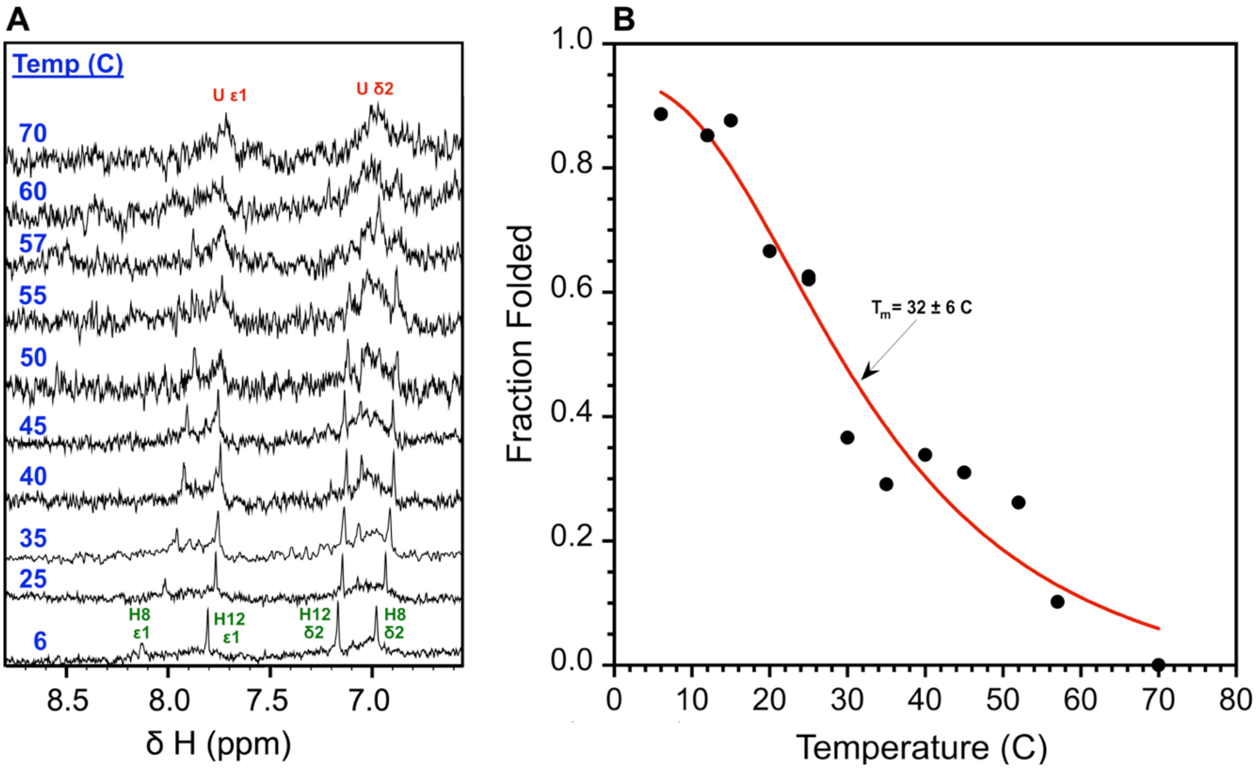

Supplementary Figure S4 Thermal unfolding of ZL-Zn^{2+} by NMR (**A**) Thermal melt monitored by 1d ^{1}H-NMR spectra of the aromatic resonances from the two histidines in ZL-Zn^{2+}. The sample was 300 μM ZL-Zn^{2+} at pH 6.9. Each of the two histidines gives resolved Hε1 and Hδ2 signals in the folded state (green). With increasing temperature, the Hε1 signal from the unfolded state of both histidines (red, Uε1) overlaps with the folded Hε1 signal for H12. The unresolved signal from the Hδ2 resonances of the two histidines in the unfolded state (red, Uδ2) is positioned between the two folded-state Hδ2 resonances and was used to calculate the fraction of unfolded protein at each temperature using the integration routine of the program iNMR. (**B**) Fitting of the fraction of folded protein versus temperature to a sigmoidal curve gave a midpoint for thermal unfolding of 32 ± 6 C.

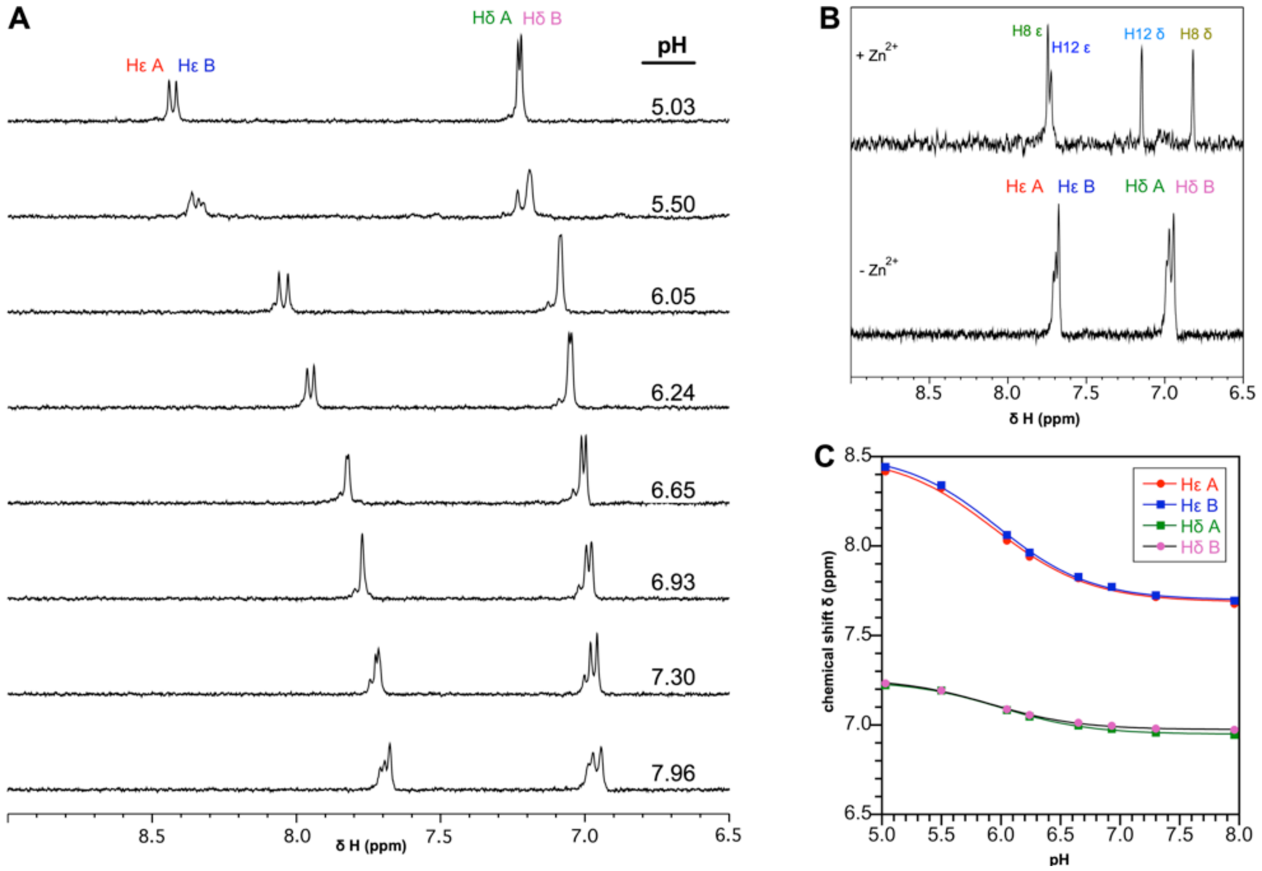

Supplementary Figure S5 Histidine pH titrations for the unfolded ZL fragment without zinc. (**A**) NMR pH titration at 600 MHz of the ZL peptide in the absence of zinc, for comparison with the data in Fig. 4B for the folded zinc-bound peptide. Because the histidines are unassigned in the unfolded state, we label them HA and HB. (**B**) Comparison of the aromatic regions of ^{1}H NMR spectra for the ZL peptide with and without Zn^{2+} for samples in D_2O at pH 8.0, showing the differences in histidine chemical shifts accompanying zinc-induced folding. (**C**) Fitting of the pH titration data to the Henderson-Hasselbalch equation gives pK_a values of 5.96 ± 0.02 and 5.95 ± 0.06 for the two histidines in the unfolded state. For comparison, in the zinc-bound folded state the pKa values were 6.9 ± 0.1 and 5.5 ± 0.1 for His8 and His 12, respectively (Fig. 4).

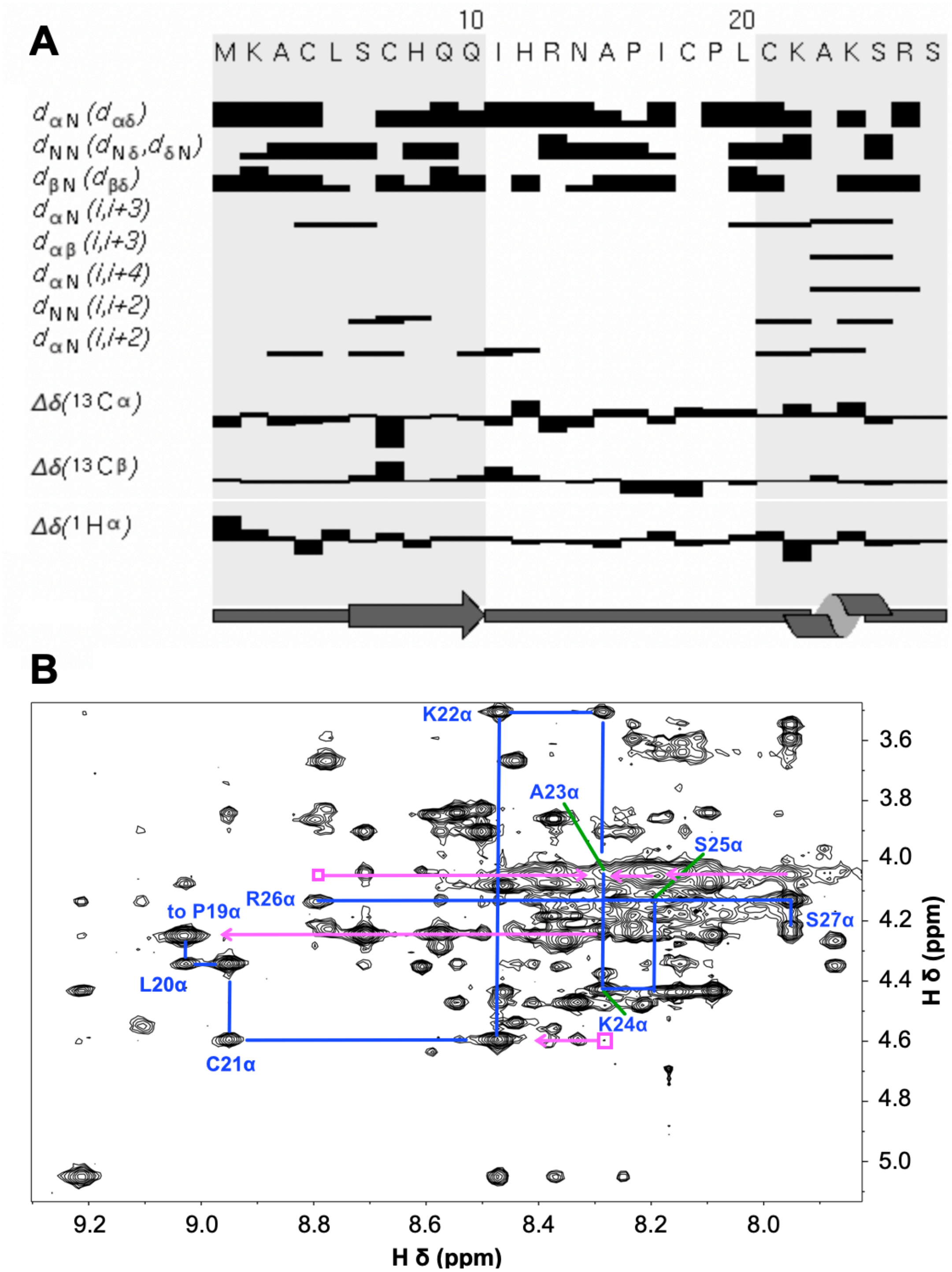

Supplementary Figure S6 NMR secondary structure and NOEs supporting a short C-terminal α-helix (**A**) Wüthrich diagram showing short-range NOEs, chemical shift indices, and consensus NMR-derived secondary structure. (**B**) NOEs supporting a short one-turn α-helix at the C-terminus. Labels indicate intraresidue HN-Hα NOEs, blue lines trace sequential NOEs, and pink lines non-sequential inter-residue HN-Hα NOEs supporting α-helical structure.

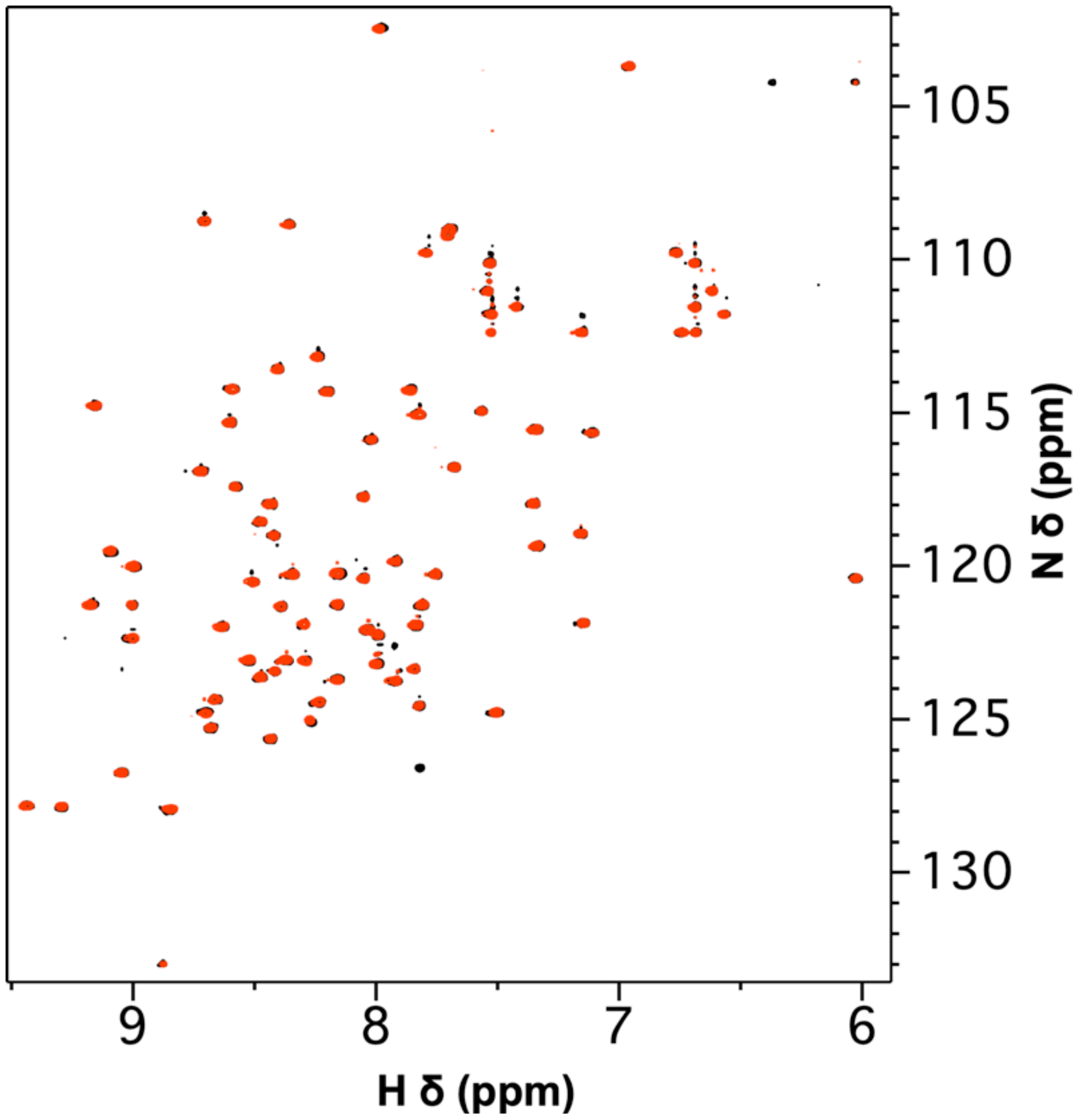

Supplementary Figure S7 NMR fails to detect interaction between ZC4H2-ZL and ubiquitin. To a 0.2 mM sample of recombinantly-produced human ^{15}N-ubiquitin in a buffer containing 150 mM NaCl and 50 mM phosphate, pH 7; we added 0.5, 1.0, 2.0, and 4.0 molar ratios of ZL-Zn^{2+} from a 9.6 mM stock solution. Data were recorded on a Varian Inova 600 MHz spectrometer at a temperature of 25 C. None of the ^{15}N-ubiquitin ^{1}H-^{15}N HSQC spectra showed changes upon addition of unlabeled ZL-Zn^{2+}. The ^{1}H-^{15}N HSQC spectra shown in the figure compare ubiquitin alone (black) to 4:1 ubiquitin: ZL-Zn^{2+} (red). The nearly complete superposition of the two spectra indicates that the two proteins do not interact.

Table S1 – Statistics for the 20 best NMR structures of the ZL fragment.

NMR restraints (total)	252	
Distance (total) [a]	206	
Intraresidue NOE ($\|i-j\| = 0$)	33	
Sequential NOE ($\|i-j\| = 1$)	77	
Medium-range NOE ($1<\|i-j\|<5$)	23	
Long-range NOE ($\|i-j\|\geq 5$)	49	
Hydrogen bond [b]	10	
Zn^{2+} coordination restraints [c]	14	
Dihedral (ϕ 19, ψ 19, χ_1 8)	46	
Residual restraint violations		
Distance (Å)	0.0471 ± 0.0032 [d]	
Dihedral (°)	1.07 ± 0.14	
RMS deviations from ideal geometry		
Bonds (Å)	0.00467 ± 0.00020	
Angles (°)	0.75 ± 0.06	
Improper torsions (°)	2.60 ± 0.31	
Ramachandran plot PROCHECK statistics for ordered residues		
Residues in most favored regions	73.8 %	
Residues in allowed regions	21.2 %	
Residues in generously allowed regions	5.0 %	
Residues in disallowed regions	0.0 %	
Coordinate rms deviations (Å)	Backbone	All
NMR ensemble to mean	0.69 ± 0.28	1.39 ± 0.40
NMR ensemble to mean (ordered: K2-H8, Q10-S25)	0.60 ± 0.25	1.22 ± 0.36

[a]The PdbStat program was used to remove redundant and structurally non-informative NOEs from the final restraints described in this table.
[b]Two distance restraints (1.5-2.5 Å for NH-O and 2.5-3.5 Å for N-O) for each of five hydrogen bonds were included to enforce hydrogen bond linearity.
[c]Two restraints per Cys-Zn^{2+} bond (from Sγ and Cβ to Zn^{2+}) were used to maintain linearity for a total of eight, and an additional six Sγ-Sγ restraints between each Cys-Cys pair combination were used to enforce tetrahedral geometry.
[d]All values are given as mean ± standard deviation.
